# Pain catastrophizing and its association with functional disability and psychological distress in patients with chronic low back pain: a retrospective study

**DOI:** 10.3389/fmed.2026.1735241

**Published:** 2026-03-18

**Authors:** Deng-Peng Wen, Xiao Liu, Tian Gan, Meng-Lin Zeng, Shao-Jun Wu

**Affiliations:** 1 Department of Rehabilitation, Dazhou Dachuan District People's Hospital (Dazhou Third People's Hospital), Dazhou, Sichuan, China; 2 Department of Tuina, Hospital of Chengdu University of Traditional Chinese Medicine, Chengdu, Sichuan, China; 3 Department of Orthopedic, Dazhou Dachuan District People's Hospital (Dazhou Third People's Hospital), Dazhou, Sichuan, China

**Keywords:** chronic low back pain, depressive symptoms, functional disability, pain catastrophizing, psychological factors

## Abstract

**Background:**

Chronic low back pain (CLBP) is a leading cause of disability worldwide, with growing recognition of the role of psychological factors in its persistence and severity. Pain catastrophizing—a maladaptive cognitive-emotional response to pain—has been linked to greater pain intensity and poor functional outcomes. However, evidence from real-world clinical settings remains limited.

**Methods:**

This retrospective study included 392 patients with CLBP treated at Dazhou Dachuan District People's Hospital between January 2018 and December 2023. Pain intensity, catastrophizing, depressive and anxiety symptoms, kinesiophobia, sleep quality, and disability were assessed using standardized scales (NRS, PCS, PHQ-9, GAD-7, TSK-11, PSQI, and ODI). Spearman correlation and multivariable linear regression analyses were performed to identify factors associated with functional disability, and a conceptual mediation model was tested to evaluate the indirect effect of depression. Mediation testing was conceptual and based on cross-sectional associations within a retrospective dataset; therefore, temporal ordering and longitudinal mediation cannot be inferred.

**Results:**

Pain catastrophizing was strongly correlated with ODI scores (ρ = 0.68, *p* < 0.001) and remained an independent predictor of disability after adjustment for pain intensity, depression, anxiety, and demographics (β = 0.62, *p* < 0.001). Depressive symptoms partially mediated the relationship between catastrophizing and disability. Patients in higher PCS quartiles showed progressively worse ODI scores (*p* < 0.001).

**Conclusion:**

Pain catastrophizing is a robust independent predictor of functional disability in patients with CLBP, and its effect is partly mediated by depressive symptoms. Routine psychological screening and targeted cognitive-behavioral interventions may improve functional outcomes and quality of life in this population. Given the retrospective design, these findings should be interpreted as associations rather than evidence of causality.

## 1 Introduction

Chronic low back pain (CLBP) is one of the leading causes of disability worldwide, contributing substantially to reduced quality of life and socioeconomic burden (1–3). Despite advances in pain management and rehabilitation, many patients continue to experience persistent pain and activity limitation, suggesting that biological factors alone cannot fully explain the chronicity and severity of symptoms. Accumulating evidence indicates that psychological and behavioral processes play a pivotal role in the development and maintenance of chronic pain (4, 5).

Among these factors, pain catastrophizing—a maladaptive cognitive-emotional response characterized by rumination, magnification, and helplessness—has attracted increasing attention in recent years (6). Patients with higher catastrophizing levels tend to report greater pain intensity, emotional distress, and disability, regardless of objective pathology (7). Pain catastrophizing has been linked to enhanced central sensitization, hypervigilance to pain cues, and impaired descending inhibitory control, thereby amplifying the pain experience and hindering functional recovery (8, 9). Previous studies have shown that catastrophizing interacts with depression, anxiety, and sleep disturbances, forming a complex biopsychosocial loop that sustains disability in CLBP (10, 11). Among these factors, pain catastrophizing—a maladaptive cognitive-emotional response characterized by rumination, magnification, and helplessness—has attracted increasing attention in recent years (12). Patients with higher catastrophizing levels tend to report greater pain intensity, emotional distress, and disability, regardless of objective pathology (13, 14). Pain catastrophizing has been linked to enhanced central sensitization, hypervigilance to pain cues, and impaired descending inhibitory control, thereby amplifying the pain experience and hindering functional recovery (15, 16). Previous studies have shown that catastrophizing interacts with depression, anxiety, and sleep disturbances, forming a complex biopsychosocial loop that sustains disability in CLBP (17). Nevertheless, evidence from routine rehabilitation settings remains limited, and the extent to which psychological distress may statistically account for the catastrophizing–disability association is not well characterized. Accordingly, we examined the association between pain catastrophizing and functional disability in a real-world clinical rehabilitation sample and explored a conceptual, cross-sectional pathway in which psychological distress may partially explain this association.

Therefore, this retrospective study aimed to investigate the relationship between pain catastrophizing and functional disability in patients with CLBP, using routinely collected clinical and psychological data from a hospital-based cohort. We further explored whether depressive symptoms partially mediate this association. Clarifying these relationships may help clinicians identify high-risk patients and develop more comprehensive, psychologically informed rehabilitation strategies for CLBP.

## 2 Methods

### 2.1 Study design and participants

This retrospective observational study was conducted at Dazhou Dachuan District People's Hospital (Sichuan, China). Electronic medical records of patients diagnosed with CLBP between January 2018 and December 2023 were reviewed. Participants were evaluated in the rehabilitation medicine department of our institution, including assessments conducted during outpatient visits and, when applicable, during inpatient hospitalization.

Inclusion criteria were: (1) diagnosis of CLBP persisting for more than 3 months; (2) availability of complete data on pain intensity, psychological questionnaires, and functional assessment; and (3) age between 18 and 85 years.

Exclusion criteria were: (1) history of acute spinal trauma, tumor, or infection; (2) severe psychiatric or cognitive disorders preventing questionnaire completion; or (3) missing key clinical or imaging data.

Missing data management. A total of 430 eligible patients were initially identified, and after excluding incomplete records, 392 participants were included in the final analysis. Incomplete records were defined as missing values in one or more key variables required for the prespecified analyses (patient-reported questionnaires and core covariates), and thus these cases were not eligible for inclusion in the primary regression models. We explored patterns of missingness by comparing available baseline characteristics between included and excluded cases and did not observe clear evidence that exclusions were concentrated within a specific demographic subgroup. However, because this was a retrospective study based on routinely collected clinical data, the missingness mechanism (MCAR vs. MAR vs. MNAR) cannot be definitively established, and no formal MCAR testing was performed. Accordingly, complete-case analysis may introduce selection bias if missingness is related to pain catastrophizing, functional disability, or psychological distress; this potential impact has been acknowledged in the limitations, and all findings are interpreted as associations within the available clinical records.

Because this study was retrospective and based solely on anonymized clinical data, ethical approval was waived by the Institutional Review Board of Dazhou Dachuan District People's Hospital, in accordance with the Declaration of Helsinki. All data were de-identified before analysis, and no intervention or additional patient contact was involved.

### 2.2 Clinical and psychological assessments

Pain intensity was evaluated using the Numeric Rating Scale (NRS), a self-reported 11-point scale ranging from 0 (“no pain”) to 10 (“worst imaginable pain”). Pain catastrophizing was assessed using the Pain Catastrophizing Scale (PCS), consisting of 13 items rated on a 5-point Likert scale (0–4), with total scores ranging from 0 to 52; higher scores indicate stronger catastrophizing tendencies. Depressive symptoms were assessed using the Patient Health Questionnaire-9 (PHQ-9; range 0–27). Higher scores indicate more severe depressive symptoms; scores of 5, 10, 15, and 20 correspond to mild, moderate, moderately sev ere, and severe symptom levels, respectively. A score of ≥10 was considered a clinically relevant threshold indicating at least moderate depressive symptoms. Anxiety symptoms were assessed using the Generalized Anxiety Disorder-7 (GAD-7; range 0–21), with cut points of 5, 10, and 15 representing mild, moderate, and severe anxiety, respectively; a score of ≥10 was considered clinically relevant. In the primary regression analyses, PHQ-9 and GAD-7 were entered as continuous variables to preserve information and statistical power.

Kinesiophobia, or fear of movement, was assessed using the 11-item Tampa Scale for Kinesiophobia (TSK-11), with scores from 11 to 44; higher values reflect stronger fear-avoidance beliefs. Sleep quality was measured using the Pittsburgh Sleep Quality Index (PSQI), ranging from 0 to 21, where higher scores indicate poorer sleep. Functional disability related to LBP was quantified using the Oswestry Disability Index (ODI), which ranges from 0 to 100; higher scores represent greater disability severity. Physical mobility was assessed using the Timed Up and Go (TUG) test. Participants were instructed to stand up from a chair, walk 3 meters at a comfortable and safe pace, turn, return to the chair, and sit down. The time (in seconds) required to complete the task was recorded; longer times indicate poorer functional mobility.

Comorbidities and medication use were not systematically captured in a standardized manner across records and were therefore not included in the primary analyses. All scales were administered and recorded by trained rehabilitation clinicians during routine outpatient assessments. Each questionnaire was completed by the patient independently, with assistance provided when necessary to ensure comprehension.

### 2.3 Imaging evaluation

All participants underwent lumbar magnetic resonance imaging (MRI) as part of their routine clinical evaluation. Sagittal and axial T1- and T2-weighted images were reviewed independently by two experienced radiologists who were blinded to the patients' psychological and functional data. The degree of intervertebral disc degeneration was classified according to the Pfirrmann grading system (Grades I–V), based on nucleus pulposus signal intensity, disc structure, and disc height. Grades I–II were defined as mild degeneration, Grade III as moderate, and Grades IV–V as severe degeneration. The presence of disc herniation was recorded as present or absent (including protrusion and extrusion), defined as displacement of nucleus material beyond the intervertebral space. When discrepancies arose between the two radiologists, a consensus decision was reached after joint review. In addition, nerve root compression and Modic endplate changes were documented when available.

Importantly, MRI parameters were collected for clinical characterization but were not included as predictors in the primary regression models. This decision was made because degenerative imaging findings in chronic low back pain do not consistently correspond to symptom severity or functional disability, and because routine radiology documentation may be heterogeneous and not uniformly available in a standardized format for modeling. To avoid unnecessary loss of analyzable cases and to prioritize model stability and comparability across participants, the primary analyses focused on patient-reported measures and core demographic/clinical covariates.

### 2.4 Statistical analysis

All statistical analyses were performed using SPSS version 26.0 (IBM Corp., Armonk, NY, USA) and R software version 4.3.2 (R Foundation for Statistical Computing, Vienna, Austria). Continuous variables were expressed as mean ± standard deviation (SD) or median (interquartile range, IQR) depending on distribution, and categorical variables were summarized as counts and percentages. For descriptive comparisons, patients were categorized into low and high PCS groups based on the median PCS score of the analytic sample (PCS Low: below the median; PCS High: at or above the median). Group comparisons were conducted using the independent samples *t*-test or Mann–Whitney *U*-test for continuous variables and the χ^2^ test or Fisher's exact test for categorical variables, as appropriate. In addition, PCS scores were also categorized into quartiles (Q1–Q4; approximately 25% of participants per quartile) as a complementary analysis to explore potential dose–response patterns across increasing levels of catastrophizing, as reported in the Results.

Spearman's rank correlation analyses were used to quantify relationships among key clinical and psychological variables, including PCS, ODI, NRS, PHQ-9, GAD-7, TSK-11, and PSQI, and are reported as correlation coefficients (r) with corresponding *p*-values. To identify independent predictors of functional disability, a multivariable linear regression model was constructed with ODI as the dependent variable and PCS, NRS, PHQ-9, GAD-7, age, BMI, sex, and radiculopathy as covariates. Model assumptions were evaluated by assessing multicollinearity (variance inflation factor < 5), residual normality, and homoscedasticity. Additionally, an exploratory conceptual mediation analysis was performed to examine whether PHQ-9 statistically accounted for part of the association between PCS and ODI; given the retrospective cross-sectional design, this analysis was interpreted as hypothesis-generating rather than causal. All statistical tests were two-tailed, and a *p*-value < 0.05 was considered statistically significant.

## 3 Results

### 3.1 Baseline characteristics

A total of 392 patients with CLBP were included in the final analysis. Participants were stratified into low catastrophizing (PCS ≤ 19, *n* = 196) and high catastrophizing (PCS > 19, *n* = 196) groups. The mean age of the cohort was 51.3 ± 19.3 years, and 59.2% were female. The mean BMI was 26.4 ± 4.6 kg/m^2^, and the median duration of LBP was approximately 27 months (IQR 12–48). Sex distribution did not differ significantly between the PCS Low and PCS High groups (*p* = 0.759) (Table 1).

**Table 1:** Baseline characteristics of patients stratified by pain catastrophizing level.

**Variable**	**PCS low**	**PCS high**	***P*-value**
Age	51.7 ± 19.8	51.2 ± 18.8	0.841
BMI	26.7 ± 4.9	26.2 ± 4.2	0.289
Duration months	30.5 ± 35.1	28.3 ± 30.1	0.886
Pain NRS (0–10)	2.8 ± 1.6	4.4 ± 1.6	< 0.001
PHQ-9 (0–27)	6.0 ± 3.9	9.7 ± 4.3	< 0.001
GAD-7 (0–21)	5.1 ± 3.5	7.7 ± 3.6	< 0.001
TSK-11 (11–44)	22.1 ± 4.7	26.8 ± 4.6	< 0.001
PSQI (0–21)	5.3 ± 2.2	7.3 ± 2.3	< 0.001
ODI (0–100)	14.2 ± 10.1	27.3 ± 11.8	< 0.001
TUG (s)	10.9 ± 2.1	11.5 ± 1.9	0.002
Tender	3.8 ± 2.7	5.0 ± 2.9	< 0.001
Sex			0.759
Male	120 (54.1%)	95 (55.9%)	
Famale	102 (45.9%)	75 (44.1%)
Education			0.207
Primary	79 (37.8%)	47 (29.0%)
Secondary	37 (17.7%)	33 (20.4%)
College+	93 (44.5%)	82 (50.6%)
Smoking			0.191
Never	44 (21.7%)	38 (23.5%)
Former	65 (32.0%)	38 (23.5%)
Current	94 (46.3%)	86 (53.1%)
Alcohol			0.272
None	11 (5.2%)	15 (9.1%)	
Moderate	129 (60.8%)	91 (55.5%)	
Heavy	72 (34.0%)	58(35.4%)	
Hypertension	152 (68.5%); 70 (31.5%)	115 (67.6%); 55 (32.4%)	0.913
Diabetes	189 (85.1%); 33 (14.9%)	148 (87.1%); 22 (12.9%)	0.660
Radiculopathy	158 (71.2%); 64 (28.8%)	108 (63.5%); 62 (36.5%)	0.127
MRI disc degeneration grade			0.489
1	45 (20.3%)	28 (16.5%)	
2	47 (21.2%)	30 (17.6%)	
3	50 (22.5%)	39 (22.9%)	
4	40 (18.0%)	42 (24.7%)	
5	40 (18.0%)	31 (18.2%)	
MRI herniation			1
0	133 (59.9%)	102 (60.0%)	
1	89 (40.1%)	68 (40.0%)	
Opioid			0.338
0	202 (91.0%)	160 (94.1%)	
1	20 (9.0%)	10 (5.9%)	
NSAID			0.683
0	106 (47.7%)	77 (45.3%);	
1	116 (52.3%)	93 (54.7%)	
SLR positive			0.308
0	164 (73.9%);	117 (68.8%);	
1	58 (26.1%)	53 (31.2%)	
Disc degeneration			0.008
Mild (I–II)	80 (36.0%)	41 (23.5%)	
Moderate (III)	90 (40.5%)	69 (41.2%)	
Severe (IV–V)	52 (23.4%)	60 (35.3%)	
Disc herniation			0.004
0	132 (59.5%)	75 (44.1%)	
1	90 (40.5%)	95 (55.9%)	

Baseline characteristics of participants by PCS group. Data are presented as mean ± SD for continuous variables and n (%) for categorical variables. *P*-values were calculated using Student's *t*-test or Mann–Whitney *U*-test for continuous variables and χ^2^ test or Fisher's exact test for categorical variables, as appropriate. PCS, Pain Catastrophizing Scale; ODI, Oswestry Disability Index; NRS, Numeric Rating Scale; PHQ-9, Patient Health Questionnaire-9; GAD-7, Generalized Anxiety Disorder-7; TSK-11, Tampa Scale of Kinesiophobia-11; PSQI, Pittsburgh Sleep Quality Index; TUG, Timed Up and Go.

Patients in the high catastrophizing group showed significantly higher pain intensity (NRS: 4.4 ± 1.6 vs. 2.8 ± 1.6, *p* < 0.001), higher depressive (PHQ-9) and anxiety (GAD-7) scores (*both p* < 0.001), and elevated kinesiophobia (TSK-11) and poor sleep quality (PSQI) compared to the low catastrophizing group (both *p* < 0.001). Furthermore, functional disability (ODI) was markedly worse in the high catastrophizing group (27.3 ± 11.8 vs. 14.2 ± 10.1, *p* < 0.001).

There were no significant differences between groups regarding age, BMI, or disease duration (all *p* > 0.05). Objective function, as reflected by TUG test performance, was also slower in the high PCS group (*p* = 0.002). Other clinical characteristics, including comorbidities, medication use, and MRI findings, were comparable between groups (all *p* > 0.05).

### 3.2 Correlation analysis

**Figure 1:**
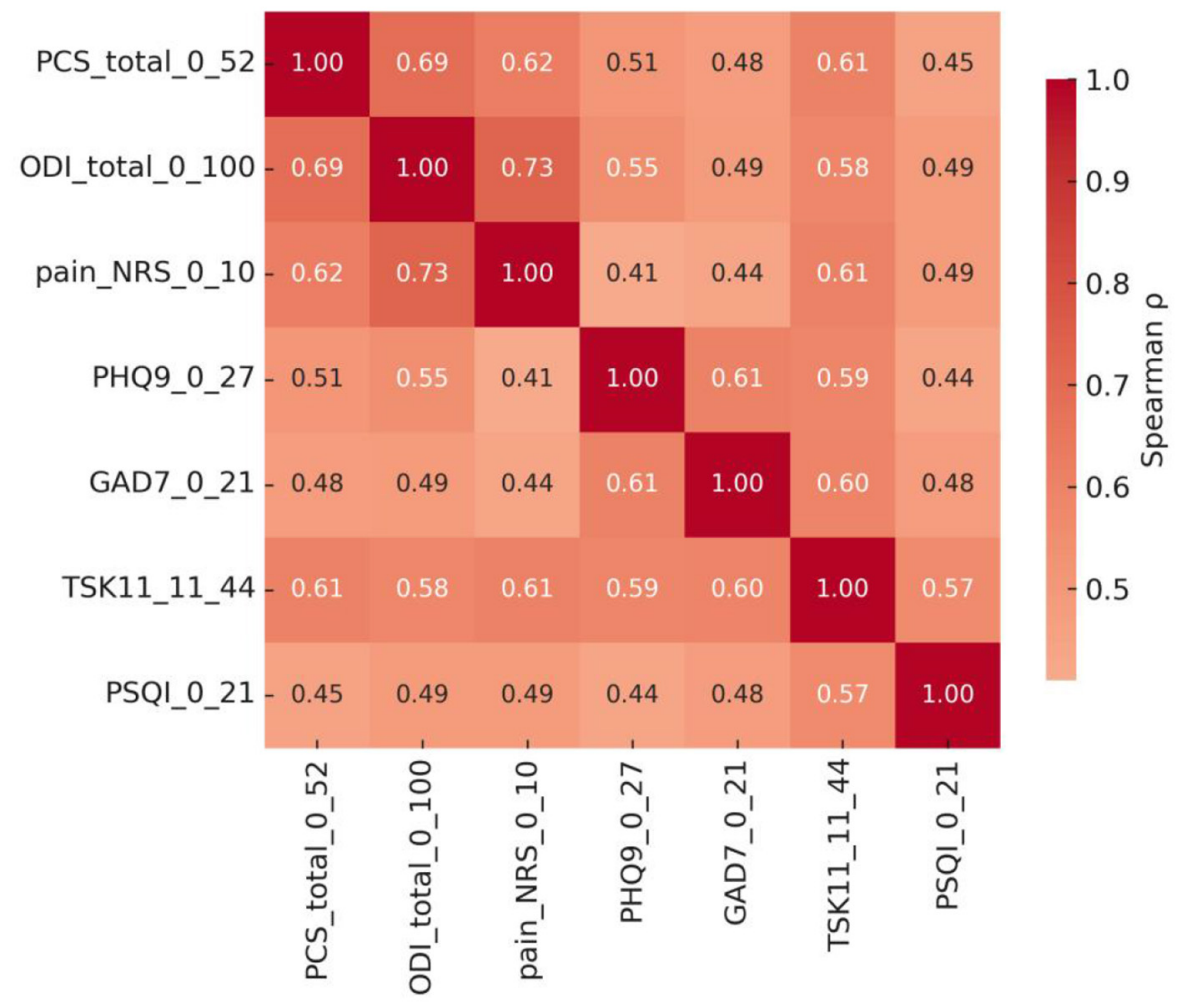
Visualizes these associations, showing a cluster of strong positive correlations linking PCS, pain, disability, and psychological variables, suggesting a shared affective-cognitive pathway underlying CLBP.

Spearman's rank correlation analyses demonstrated significant associations between pain catastrophizing (PCS) and key clinical and psychological variables (Figure 1). PCS scores were positively correlated with functional disability (ODI; *r* = 0.68, *p* < 0.001) and pain intensity (NRS; *r* = 0.59, *p* < 0.001). PCS was also positively correlated with depressive symptoms (PHQ-9; *r* = 0.63, *p* < 0.001), anxiety symptoms (GAD-7; *r* = 0.57, *p* < 0.001), kinesiophobia (TSK-11; *r* = 0.54, *p* < 0.001), and poor sleep quality (PSQI; *r* = 0.49, *p* < 0.001). Pain intensity (NRS) was positively correlated with ODI (*r* = 0.62, *p* < 0.001). Overall, higher catastrophizing levels were associated with greater pain intensity, worse functional disability, and greater psychological distress.

### 3.3 Multivariable regression analysis

To determine whether pain catastrophizing independently predicted functional disability, a multivariable linear regression model was constructed with the ODI as the dependent variable and PCS, NRS, depression (PHQ-9), anxiety (GAD-7), age, BMI, sex, and radiculopathy as covariates (Table 2). The overall model was significant [*F*_(8, 383)_ = 66.1, *p* < 0.001], explaining approximately 58% of the variance in ODI scores (adjusted R^2^ = 0.58). After adjustment for all covariates, PCS remained independently associated with functional disability (B = 0.45, 95% CI 0.28–0.62; *p* < 0.001).

**Table 2:** Multivariable linear regression predicting ODI.

**Variable**	**Coef**.	**SE**	**β**	***t***	***P*-value**	**95%CI**
Intercept	−13.62	3.00	–	−4.54	< 0.001	[−19.53, −7.71]
PCS	0.45	0.09	0.18	5.18	< 0.001	[0.28, 0.62]
Pain NRS	3.62	0.32	0.60	11.30	< 0.001	[2.99, 4.25]
PHQ	0.61	0.12	0.21	5.25	< 0.001	[0.38, 0.83]
GAD	0.07	0.14	0.02	0.48	0.63	[−0.21, 0.34]
Age	0.11	0.02	0.18	5.22	0.00	[0.07, 0.15]
BMI	0.05	0.09	0.02	0.59	0.56	[−0.12, 0.23]
Radiculopathy	−0.05	0.95	−0.01	−0.05	0.96	[−1.92, 1.83]
Sex	0.27	0.82	0.01	0.33	0.74	[−1.35, 1.89]

**Figure 2:**
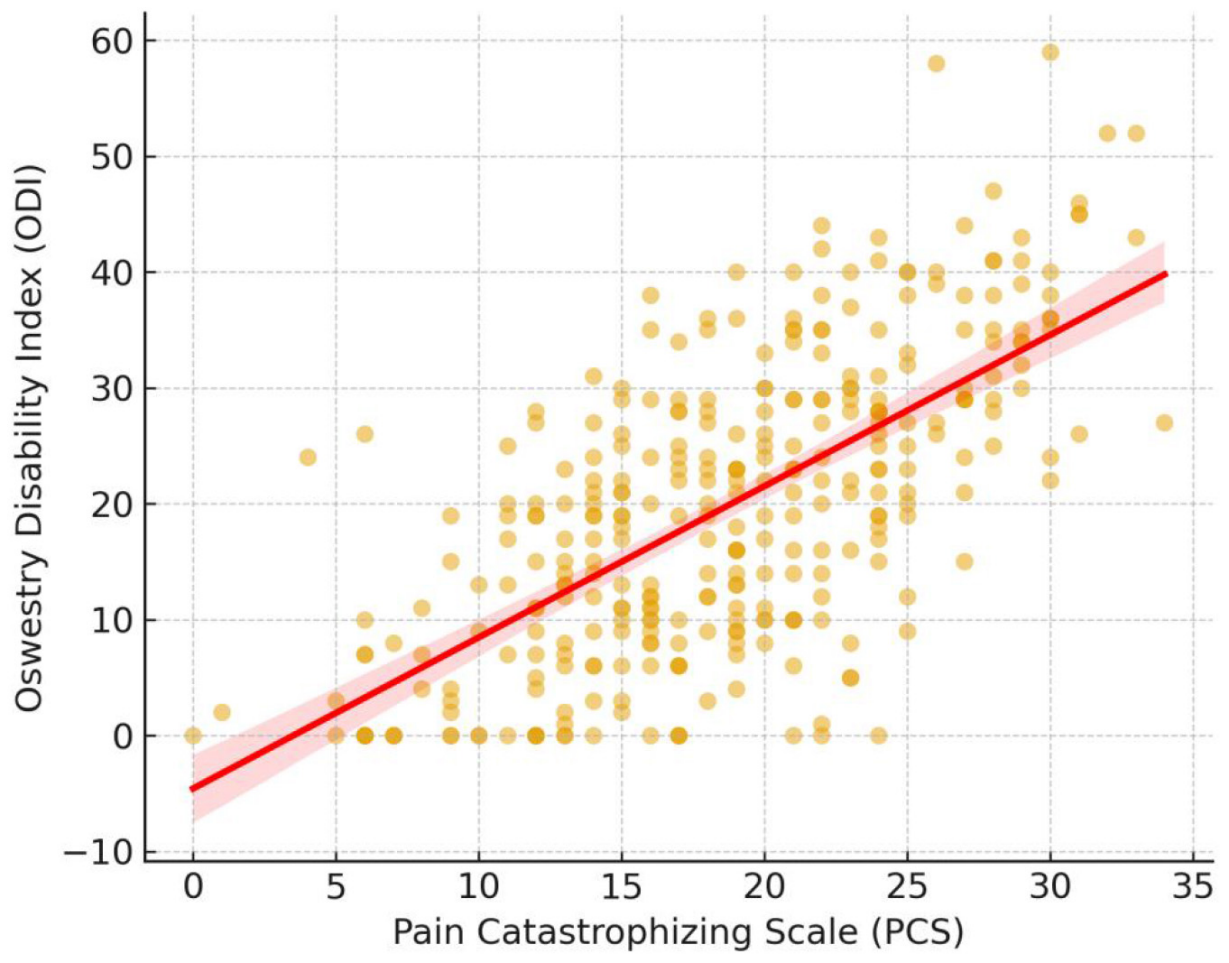
Association between PCS and ODI.

Higher PCS scores were associated with greater functional limitation on the ODI (Figure 2). Pain intensity (β = 0.29, *p* < 0.001^*^) and depressive symptoms (β = 0.18, *p* < 0.01^*^) were also significant independent correlates of disability, whereas anxiety, age, BMI, sex, and radiculopathy did not reach statistical significance (all *p* > 0.05).

These results indicate that pain catastrophizing contributes uniquely and substantially to disability among patients with CLBP, independent of physical pain intensity or depressive symptom burden.

### 3.4 Subgroup and mediation analyses

**Figure 3:**
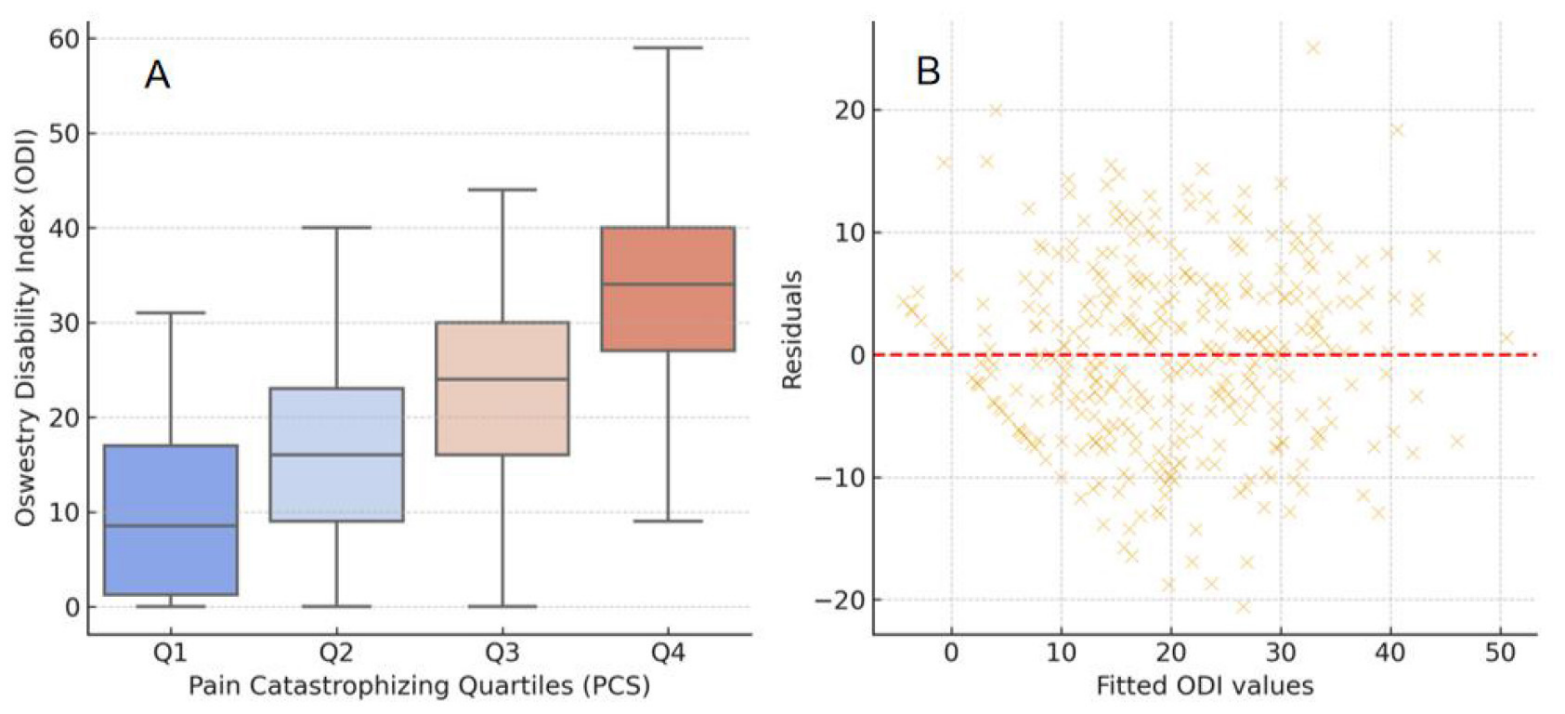
Comprehensive visualization of the relationship between pain catastrophizing and functional disability. **(A)** Comparison of ODI scores across quartiles of PCS; ODI increased progressively with higher PCS levels (*p* < 0.001). **(B)** Residual plot showing the distribution of fitted vs. residual values, indicating good model fit and homoscedasticity.

In addition to the median-based PCS Low/High grouping used for descriptive comparisons, we performed a quartile-based analysis (Q1–Q4) to examine potential dose–response patterns across increasing PCS levels (Figure 3A). A progressive increase in ODI scores was observed across PCS quartiles (*p* < 0.001, Kruskal–Wallis test), indicating that higher catastrophizing was consistently associated with greater disability. Residual analysis of the multivariable regression model (Figure 3B) showed a random distribution of residuals around zero without obvious heteroscedasticity, supporting the adequacy of the fitted model.

A conceptual mediation model was then examined to explore potential psychological pathways. Spearman correlations suggested that depressive symptoms (PHQ-9) might partially mediate the relationship between PCS and ODI, with significant associations observed between PCS and PHQ-9 (*r* = 0.63, *p* < 0.001), PHQ-9 and ODI (*r* = 0.67, *p* < 0.001), and PCS and ODI (*r* = 0.68, *p* < 0.001). These findings support the hypothesis that pain catastrophizing contributes to functional disability partly through increased depressive symptomatology.

## 4 Discussion

Relevance to real-world rehabilitation practice. A key motivation for this study was the limited evidence describing pain catastrophizing and disability in routine rehabilitation settings. Our findings extend prior work by demonstrating that higher PCS scores are robustly associated with greater functional disability in a real-world clinical rehabilitation sample, where comorbid symptoms and heterogeneity in presentations are common. This pragmatic context strengthens the clinical interpretability of catastrophizing screening and supports its integration into rehabilitation pathways, while still acknowledging that the retrospective cross-sectional design does not establish causality.

In this retrospective study of 392 patients with CLBP, we found that pain catastrophizing was strongly associated with functional disability, even after adjusting for pain intensity, depressive and anxiety symptoms, and demographic factors. Higher PCS scores were consistently related to greater disability on the ODI, and this relationship remained robust in multivariable analyses. Furthermore, depressive symptoms partially mediated the association between pain catastrophizing and disability, suggesting that emotional distress contributes to the functional consequences of maladaptive pain cognitions. These results highlight the pivotal role of psychological factors in chronic pain–related disability and emphasize the need for integrative rehabilitation strategies that address both physical and psychological domains.

Our findings align with previous research demonstrating that pain catastrophizing is a critical psychological determinant of pain severity and disability in CLBP (18). Smeets et al. (19) first described catastrophizing as an exaggerated negative mental set toward pain experiences, which amplifies perceived pain and hinders adaptive coping. Subsequent studies have confirmed its predictive value for treatment outcomes and return-to-work rates in chronic musculoskeletal conditions (20). Consistent with these results, our study revealed that catastrophizing was independently related to disability beyond the effects of pain intensity and depressive symptoms. This finding supports the biopsychosocial model of pain, in which cognitive-emotional processes influence functional outcomes through both direct and indirect pathways.

Several neurophysiological and behavioral mechanisms may underlie the observed associations. Pain catastrophizing has been linked to altered activity in brain regions such as the anterior cingulate cortex, insula, and prefrontal cortex, which are involved in pain modulation and emotional regulation (21, 22). High catastrophizing individuals tend to display enhanced pain vigilance and impaired descending inhibitory control, leading to sustained nociceptive amplification (23, 24). At the behavioral level, catastrophizing often co-occurs with avoidance behaviors, reduced physical activity, and sleep disruption—all of which contribute to deconditioning and disability (25–27). The partial mediation by depressive symptoms observed in our study suggests that catastrophizing may exacerbate emotional dysregulation, further amplifying pain-related disability. Rather than reiterating that psychological factors matter in CLBP, the present study adds pragmatic evidence from a rehabilitation clinical sample and quantifies that catastrophizing remains independently associated with disability even after accounting for pain intensity and concurrent psychological symptoms.

Clinically, these findings emphasize the importance of early identification of patients with high pain catastrophizing in rehabilitation practice. Incorporating cognitive-behavioral interventions, mindfulness-based training, and psychological counseling may enhance treatment efficacy and functional recovery. Routine assessment of PCS, PHQ-9, and related measures could provide a cost-effective strategy for tailoring individualized management plans. However, several limitations should be noted. First, the retrospective design limits causal inference, although the large sample and real-world data enhance external validity. Second, psychological assessments were based on self-reported questionnaires, which may be subject to reporting bias. Third, the study was conducted in a single center, and generalizability to other settings requires further validation. Finally, the mediation analysis was conceptual rather than longitudinal; prospective studies are needed to confirm the temporal dynamics between catastrophizing, depression, and disability.

## 5 Conclusion

This study integrates AD GWAS with peripheral-blood eQTL/mQTL resources and evaluates brain-tissue relevance using GTEx v8 and AMP-AD to prioritize OS-related regulatory candidates. Using a three-step SMR framework, we highlight key signals including KEAP1 and SIRT1, together with methylation loci such as cg20211653 (ABCA1) and cg12453748 (PRDX5), which link epigenetic regulation to transcriptional mechanisms relevant to AD. Importantly, the chromosome 19 APOE region emerged as a regulatory hub with convergent eQTL/mQTL evidence across APOE-region genes (APOE/APOC1/TOMM40), supporting a testable model in which inherited regulatory variation may couple metabolic–mitochondrial stress responses with OS amplification and downstream neurodegenerative cascades. These results motivate concrete next steps: (i) brain-region and cell-type–resolved fine-mapping/colocalization to define the most likely shared causal variants and the relevant cellular contexts, and (ii) functional assays to test whether perturbing prioritized loci (e.g., APOE-region regulation, ABCA1/CALM1-related methylation) alters oxidative stress responses, mitochondrial dysfunction, calcium homeostasis, and AD-related phenotypes in experimental models.

## Data Availability

The original contributions presented in the study are included in the article/supplementary material, further inquiries can be directed to the corresponding authors.
